# Mr. Marson, in Reply to Mr. Foge

**Published:** 1804-04-01

**Authors:** W. Marson

**Affiliations:** Worksop


### *To the Editors of the Medical and Physical Journal.*

GENTLEMEN,

I Have neither leisure nor inclination for Controversy, though I now find myself necessitated to answer Mr. Foge's letter, Vol. xi. p. 64.

From the many year's practice of Mr. F. and the particular turn of his arguments, it is pretty manifest he is a disciple of Chapman, who published a book in 1733, or thereabouts, for the use of *women midwives.* Chapman's practice was, immediately on the birth of the child to pass his hand into the uterus and deliver the placenta; he says, "I never suffer the womb to close before it is relieved of its contents, for the moment the child is born, I slip my right hand into the womb (at which time the parts, on account of their great dilatation will allow of it without force or pain) and gently assist in the extraction of the placenta."

Mr. F. is much puzzled as to the utility of the left hand to the sacrum; the woman being laid on her left side, the accoucheur, when he presses on the abdomen with the right hand, by putting the left hand on the sacrum, places the uterus in a state between pressure and resistance; the left hand too allows the practitioner to judge better of the necessary degree of pressure, and enables him the more steadily to apply it, for he is "never to lose the pressure he has gradually arrived to;" at any rate the left hand must be somewhere, and Mr. F. has not made any soreness of of the sacrum as an objection to its being placed there. Mr. F. in saying that gradually increasing a slight pressure means more than a slight pressure, is certainly true, but I believe my meaning is intelligible to your readers.

If Mr. F. had coolly and deliberately perused my paper, and not considered it as an attack on Chapman and his practice of passing the hand into the uterus, he would have found that I spoke of pressure "being otherwise of good effect," not confining it to the deliverance of the placenta; the other "advantages" of pressure then not being objected to by Mr. F. the practice remains admitted, and I repeat, that considerable pressure on the parieties of the abdomen is of good effect in every case of labour, independent of the delivery of the placenta, and that at the time I have mentioned; as to the soreness of the abdomen being an hinderance, it merely amounts to this, when the child is born the woman thinks she has sufficiently suffered, and that the heavenly moment should not be interrupted; but tell her the pressure on the abdomen is necessary, that the success of an expeditious recovery much depends on this circumstance, she will, considering it as a part of labour, neither murmur nor complain, though the soreness was to the extent depicted by Mr. F. I believe the passing the hand into the uterus would not be so readily reconciled, and the indelicacy of the practice would more than counterbalance the suffering from pressure on the sore abdomen, as represented by Mr. F.; but passing the hand is not exempt from pain on account of the irritable soreness of the parts. It would seem that there was some danger attending Mr. F's supposed soreness of the abdomen, in his saying "too often;" but it will appear rather curious that no author or lecturer, recommending pressure, should mention soreness as an objection to its use, and that Mr. F. of whose practice it has never formed a part, should make the discovery.

The next paragraph exhibits Mr. F. making free with Dr. Denman, to give the practice I recommend a fatal blow, for the way I apply pressure never causes the mischief he supposes; "he is very well assured, he says, if the knuckles, &c." He has very artfully indeed blended the "supposed" knuckles and my mode of pressure, to prove that my hand does the injury which he has represented, and then exultingly pronounces the passing of the hand to be a preferable practice. I will not dispute Mr. F's adroitness in passing the hand, nor his great success, but he seems to be aware of hazard, and tells of his lucky escapes; escapes; and it is remarkable that he should adopt nearly the same language as Chapman, to express his good fortune. Chapman says, "Nor are the examples of many women doing very well under the management of such midwives as never do pass the hand, any objection to this method, since no one was ever hurt by it, but thousands on the contrary, have suffered, nay died, by the omission thereof."

The pressure I recommend is not stated to retard the deliverance of the placenta, on the contrary must promote it; but Mr. F. insinuates that the pressure I recommend is tantamount to leaving it to the action of the uterus. I have no where said I would not pass the hand; and rather than let my patient be endangered, I would try all means in my power, but I would put off the practice till I was convinced pressure would not answer. Mr. F. has allowed that the hand may be easily passed any time, within twenty-four hours.

The forceps are very useful; but great as my address might be in the management of them, I would not officiously employ them.

Chapman observes, that "one clod being expelled, the vessels will sometimes bleed anew," which I am surprized Mr. F. has overlooked. I present it to your young readers to caution them, when called to a case as described by Mr. F. where the hæmorrhage has ceased before his arrival, and there is a return of faintings. They must not be too hasty in ascribing the faintings to existing hæmorrhage, and immediately apply pressure, for this pressure would remove the extravasated blood that had perhaps stopped the hæmorrhage; and however proper pressure might be in flooding, it would be very impolitic to hazard a hæmorrhage by it.

Mr. Foge says, he has been called to several cases where the midwife had so far neglected the woman, as to omit pressure, and leaving the atonic uterus to be filled with blood and the placenta to be excluded by the action of the uterus. Here Mr. F. has betrayed himself, and much as he has opposed pressure, it appears to "form a part of his practice;" he seems aware that it would prevent the existing hæmorrhage, and thus supersede the necessity of passing the hand to extract the placenta. In short, Mr. F. has afterwards declared that pressure is the only thing to be depended upon in all cases of flooding, not excepting the manual extraction of the placenta.

I am ready to grant the uncertainty of the expulsion after after being detained two hours, and I will add before that time shall have expired; but in admitting this, it does not convince me that in every case of labour after the child is born I should directly pass my hand into the uterus, which Mr. F's reasoning implies.

I have already mentioned the indelicacy of passing the hand, and I believe it would be attended with danger as well as difficulty, to a person so ignorant as to invert the uterus by pulling the funis; we have much more to dread from the same want of skill in passing the hand into the uterus; the inversion of the uterus is what rarely happens, but numerous are the mischiefs recorded of the passing the hand, and hurrying away the placenta. Smellie has several cases, Hunter, Denman, Lowder, &c. all have mentioned them in their Lectures.

Mr. F. has intimated, that pressure will "separate the placenta from the uterus;" it is fair then to conclude that it would urge it also through the contraction, having no resistance from the loose parietes of the abdomen or the atonic fundus uteri.

It is rather singular that Mr. F. should cite Dr. Denman's authority, to prove that pressure is seldom or never requisite, after condemning his practice as bad.

There seems much to admire in the wariness of Mr. Barlow; but, as he is so well able to answer for himself, I shall wave saying any thing on Mr. F's disapprobation of his practice, further than making a single observation. Mr. F. says, "I am one of those who can see no safety in allowing the placenta to remain four hours under the fore-mentioned circumstances, I mean, an existing though not an apparent hæmorrhage, attended with great weakness and fainting." In an apparent hæmorrhage he would let the placenta remain four hours, but he has neglected to assign a reason for so doing.

I cannot help thinking Mr. F. culpable in secluding from the world his practice of pressure in uterine hæmorrhage; and as we are by no means certain that he will make it public, I will venture to suppose what he means: The pressure he proposes is, in all probability, by means of ligatures about the extremities, which is practised by some; and with this conjecture I shall close the subject.

I am, &c.

W. MARSON.

*Worksop, Feb.* 17, 1804.

