# Erratum

**Published:** 1804-05-01

**Authors:** 


### ERRATUM.

Page 331, l. 24, for *Barlow* read *Bartley*.

